# Reviews of Dental Literature

**Published:** 1902-03

**Authors:** 


### Reviews of Dental Literature.

---

The Disinfection of Dental Instruments by Spirits of Soap. By W. D. Miller, Berlin.[1]

Professor Miller refers to his previously published work upon various so-called antiseptics, and their value in the sterilization of dental and surgical instruments. From these investigations his conclusion was that the use of a five per cent. solution of lysol was the best chemical means for the sterilization of hands and instruments. Later other means for accomplishing this purpose have come into notice. Among these are formalin and spirit of soap. Spirit of soap has been especially recommended for the sterilization of the hands.

Inasmuch as lysol possessed a disagreeable odor, Professor Miller was interested to determine whether the spirit of soap possessed equal sterilization power to that of lysol. His experiments show that spirit of soap has far less sterilizing power than lysol, and his conclusion is that it is valuable in the treatment of the hands, but that five per cent. lysol solution is best for instruments. In the use of lysol, such instruments as are fairly smooth are immersed in it for at least half an hour. Burs are immersed from one to four hours.

The author states that in all cases where there are affections of the mucous membrane of an infectious nature, especially where there are indications that the patient has syphilis, we should sterilize the instruments in boiling water to which two per cent. of soda has been added to prevent rust. In a foot-note the author adds that since sending in the above communication (in the beginning of July) he has entirely gone over to the boiling process.

Actinomycosis.—Barclay (*Bristol Medico-Chirurgical Journal,* March, 1901) has no doubt that actinomycosis is still regarded by many as rare and infrequent, in spite of the records. He thinks that because of the obscure and ill-defined symptomatology of the

---

[1] Ueber Disinfection von zahnärztlichen Instrumenten mittelst Seifenspiritus, von W. D. Miller, Berlin. Deutsche Monatsschrift für Zahnheilkunde, 14. Dezember, 1901.

disease, and its close resemblance in clinical features to several much commoner lesions, there is much danger of failing to recognize the condition.

Once implanted, the specific organism, which grows on cereals and grasses, spreads and sets up inflammation. Sometimes an ulcer is formed, at other times a tumor of a pale-yellowish color, globular in shape, riddled with holes of a larger or smaller size containing pus, and very vascular, although it presents a superficial resemblance to the interior of a tuberculous or gummatous deposit. It infiltrates all the tissues, spreading in the intermuscular planes, and even into the muscles, entering into the cancellous tissues of bone and the solid viscera, and sometimes forming communications between the hollow viscera. The growth may be carried by the vascular channels as emboli, and deposited in distant parts of the body.

The percentage for different regions seems to be as follows: Head and neck, fifty-five per cent.; digestive tract, nineteen per cent.; pulmonary, fourteen per cent.; skin, two per cent.; and doubtful, five per cent.

In the jaw it generally simulates the common periosteal or alveolar inflammations, or occasionally suppuration in the antrum. One swelling follows another with great inveteracy, sinuses are very obstinate, and induration or infiltration is apt to occur widely in either an upward or downward direction.

Ruhräh lays great stress on the absence of involvement of the lymphatics, which he describes as a constant feature of the disease. There is an early appearance of trismus, which may be attributed to an involvement of the muscles of mastication, although in some cases the temporomaxillary joint is affected. In a case observed by Hoover during life it was impossible to show the presence of streptothrix, but at the autopsy very wide-spread disease was found.

Oliver has described a case where the disease appeared as an ulceration of the mucous membrane of the mouth. Several sores appeared in the mouth. They were curetted and healed, leaving a white indurated patch. A purulent affection of the gums appeared coincidently; the teeth were removed with the exception of two, to which artificial ones were clamped. An ulcer appeared opposite the clamp in the right cheek and spread rapidly. No streptothrix or tubercle bacilli were found in the scrapings. Iodide of potassium was given in large doses for two months, but the ulceration only extended farther and became very painful. While an operation for swellings that had appeared was being considered, the submaxillary swelling softened. On excision, a thin, greenish-yellow fluid containing some yellowish-white curdy masses was discharged. The microscope failed to show the streptothrix. A radical operation was undertaken, and several weeks later a lump under the ear softened, and on being opened the same greenish fluid was evacuated, in which, after prolonged search, the streptothrix were found. The patient finally died of arterial hemorrhage, probably from the lingual artery.

Cases of this affection involving the skin are quite rare, but Leser describes two forms,—ulceration, and a discrete nodular skin inflammation, with central cicatrization and peripheral extension, as in lupus. He considers the absence of lymphatic involvement almost pathognomonic, and he says the board-like indurations and the grossly nodular conditions which are found in these cases are not usual in the other affections. The disease may also be mistaken for rodent ulcer when it occurs in the skin.

The only absolutely diagnostic feature is the discovery of sulphur granules in the discharge from sinuses or abscesses, or in the sputum or stools. Great stress has been laid by some observers on the absence of involvement of the neighboring lymphatics in all cases. Godlee says the character of the abscess, as determined by the finger, would make him almost certain of its nature,—there are indefinite spongy walls, easily breaking before the finger and bleeding very freely. Ruhräh says the back of a patient of Black's was a seat of numerous sinuses, whose puffed and reddened openings, together with the livid intervening skin, gave a perfect picture of actinomycosis; and Volkmann, Boström, and Bernhardt believe that the reddening of the skin, turning later to a violet-blue tint, shading from the centre to the periphery, is diagnostic of the disease.

The prognosis seems to depend on the possibility of a radical extirpation of the growth, and on the presence or absence of secondary infection.

Whenever possible the diseased area should be completely excised, and Ruhräh recommends that after this, where permissible, the area should be thoroughly cauterized. The administration of iodide of potassium should always be pushed at the same time, beginning with five or ten grains three times a day, and increasing it to forty or fifty grains thrice daily. For injection into sinuses, etc., a number of substances have been employed,—tincture of iodine, carbolic acid, salicylic acid, nitrate of silver solutions, iodoform. The only hopeful treatment seems to consist in excisions, or curetting, repeated as often as necessary and as early as possible, with the internal administration of iodide of potassium up to the limit of the patient's endurance.—*Therapeutic Gazette.*

